# The 2nd International Conference on Water Resource and Environment (WRE 2016)

**DOI:** 10.1186/s12898-016-0095-7

**Published:** 2016-11-07

**Authors:** Zhouli Liu, Wei Chen, Xingyuan He, Shuai Yu, Weihang Ding, Jianjun Liu, Yuliang Wu, Kaixiang Fan, Yuanchao Li, Xinqing Zheng, Shiquan Chen, Haiqun Xie, Jie Li, Zhuomin Wang, Li Xue, Zhiyang Lie, Weilong Huang, Tongtong Zhou, Kai Gao, Tiexia Zhu, Weiteng Mao, Pei Yu, Xinfang Lv, Yunchun Li, Peixi Su, Rui Shi, Yong Jiang, Xingbin Chen, Shichu liang, Junfeng Xu, Jiwei Zhu, Zhao Zhai, Linan Zhou, Yun Le, Li Cao, Ping Lu, Zhiqian Huang, Bei Zhou, Zhirong Zheng, Shihai Lv, Chaoyang Feng, Nan Song, Yan Liu, Xinxin Lu, Yawen Fan, Jianfei Guan, Jihua Wang, Xueping Zhang

**Affiliations:** 1 Key Laboratory of Forest Ecology and Management, Institute of Applied Ecology, Chinese Academy of Sciences, 72 Wenhua Road Shenhe District, Shenyang, 110016 People’s Republic of China; 2 State Key Laboratory of Oil and Gas Reservoir Geology and Exploitation (Southwest Petroleum University), Chengdu, 610500 Sichuan China; 3 School of Geoscience and Technology, Southwest Petroleum University, Chengdu, 610500 Sichuan China; 4 Technology Center, Sichuan Coal Industry Group Limited Liability Company, Chengdu, 610091 Sichuan China; 5 School of Civil Engineering and Architecture, Southwest Petroleum University, Chengdu, 610500 Sichuan China; 6 Hainan Academy of Ocean and Fisheries Sciences, Haihou, 570100 China; 7 Third Institute of Oceanography, State Oceanic Administration, Xiamen, 361005 China; 8 College of Forestry and Landscape Architecture, South China Agricultural University, Guangzhou, 510642 China; 9 Inner Mongolia University for Nationalities, Tongliao, 028043 Inner Mongolia China; 10 Marine College, Shandong University, Weihai, 264209 China; 11 Institute of Ecology and Biodiversity, Shandong University, Jinan, 250100 China; 12 Cold and Arid Regions Environmental and Engineering Research Institute, Key Laboratory of Land Surface Process and Climate Change in Cold and Arid Regions, Chinese Academy of Sciences, Lanzhou, 730000 China; 13 College of Life Science, Guangxi Normal University, Guilin, 541004 Guangxi China; 14 Key Laboratory of Ecology of Rare and Endangered Species and Environmental Protection (Guangxi Normal University), Ministry of Education, Guilin, 541004 China; 15 Tianjin Research Institute for Water Transport Engineering, Key Laboratory of Engineering Sediment, Ministry of Transport, Tanggu, Tianjin, 300456 China; 16 State Key Laboratory of Ecological Water Conservancy in the Northwest Arid Area, Xi’an University of Technology, Xi’an, 710048 China; 17 Research Institute of Complex Engineering and Management, Tongji University, Shanghai, 200092 China; 18 China Three Gorges Corporation, Beijing, 100038 China; 19 Ji’nan City Construction Investment Group Corporation Limited, Jinan, 250101 China; 20 College of Life Science, Nankai University, Tianjin, 300071 People’s Republic of China; 21 State Environmental Protection Key Laboratory of Regional Eco-process and Function Assessment, Chinese Research Academy of Environmental Sciences, Beijing, 100012 People’s Republic of China; 22 College of Life Science and Technology, Harbin Normal University, Harbin, 150025 Heilongjiang People’s Republic of China; 23 College of Geographical Science, Harbin Normal University, Harbin, 150025 Heilongjiang China

## Abstract

01 The eco-toxicological effects of cadmium stress on five ornamental plants

Zhouli Liu, Wei Chen, Xingyuan He, Shuai Yu, Weihang Ding

02 Study on the formation and release mechanisms of hydrogen sulfide in Longtan coal mine

Jianjun Liu, Yuliang Wu, Kaixiang Fan

03 Coral bleaching in the North Reef of China’s Xisha Islands in 2014

Yuanchao Li, Xinqing Zheng, Shiquan Chen, Haiqun Xie

04 Soil chemical characteristics in a *Cunninghamia lanceolata* stand suffering from ice-snow damage

Jie Li, Zhuomin Wang, Li Xue

05 Diversity of understory vegetation was under a *Cunninghamia lanceolata* stand suffering from ice-snow damage

Zhiyang Lie, Weilong Huang, Tongtong Zhou, Li Xue

06 The effect of water, nitrogen and harvesting time on yield and biomass allocation of *Helianthus tuberosus* L.

Kai Gao, Tiexia Zhu

07 The vertical variation of microbial communities in the sediment of sea cucumber pond

Weiteng Mao, Pei Yu, Xinfang Lv, Yunchun Li

08 Indicating significances of alpine plant functional groups to environmental change

Peixi Su, Rui Shi

09 Research on the daily CH_4_ fluxes of soil in summer mangrove community at Yingluogang of Guangxi, China

Yong Jiang, Xingbin Chen, Shichu liang

10 Beach protection structures in middle and lower reaches of Yangtze River

Junfeng Xu

11 Social responsibility management of large dam project with owner as core: an empirical case in China

Jiwei Zhu, Zhao Zhai, Linan Zhou, Yun Le, Li Cao

12 The ecological function value assessment analysis of urban waterfront

Jiwei Zhu, Ping Lu, Zhiqian Huang, Bei Zhou

13 Mechanism of diurnal osmotic potential changes and adjustment of three *Caragana* species in different habitats of the Inner Mongolia Plateau

Zhirong Zheng, Shihai Lv, Chaoyang Feng

14 Biodiversity of phytoplankton and environmental influences analysis of Longfeng Wetland, China

Nan Song, Yan Liu, Xinxin Lu, Yawen Fan

15 The effect of human activities on culturable soil microbes from Yaoquan Mountain in Wudalianchi, China

Jianfei Guan, Jihua Wang, Xueping Zhang

## 01 The eco-toxicological effects of cadmium stress on five ornamental plants

### Zhouli Liu, Wei Chen, Xingyuan He, Shuai Yu, Weihang Ding

#### Key Laboratory of Forest Ecology and Management, Institute of Applied Ecology, Chinese Academy of Sciences, 72 Wenhua Road, Shenhe District, Shenyang 110016, People’s Republic of China

##### Correspondence: Xingyuan He - forestry83@hotmail.com

*BMC Ecology* 2016,** 16(Suppl 2)**:01

Cadmium (Cd) is one of the most toxic heavy metals and has recently become a global concern. The eco-toxicological effects of Cd in soil–plant system are attracting more and more attention. Now many studies are mainly related to the effects of Cd on herbs and crops, and less information is on ornamental plants. Therefore in the present study, the eco-toxicological responses of five ornamental plants (*Lonicera japonica* Thunb., *Weigela florida cv. Red Prince, Ginkgo biloba* L.*, Parthenocissus quinquefolia* and *Parthenocissus semicordata*) to Cd were investigated. Five Cd treatment concentrations (0, 5, 10, 25 and 50 mg L^−1^) were set. The results showed that the difference of physiological and biochemical characteristics in the plants under different Cd concentrations, such as antioxidant enzyme activity, chlorophyll contents and cell membrance permeability. The maintenance of high superoxide dismutase (SOD), peroxidase (POD), catalase (CAT) and ascorbate peroxidase (APX) activities in *L. japonica* was observed along with the increased Cd concentration, suggesting that the plant had better tolerance than another four plants, and had strong internal detoxification mechanisms to Cd stress. The results will provide an important scientific reference for remediation and management of Cd-contaminated soil.

**Acknowledgements:** This work was supported by the National Natural Science Foundation of China (41301340), the National Science & Technology Pillar Program (2012BAC05B05), the key project of Chinese Academy of Sciences (KFZD-SW-302-01), and the major National Science & Technology project “water pollution control and management” (2012ZX07202008) of China.

## 02 Study on the formation and release mechanisms of hydrogen sulfide in Longtan coal mine

### Jianjun Liu^1,2^, Yuliang Wu^1,3^, Kaixiang Fan^1,4^

#### ^1^State Key Laboratory of Oil and Gas Reservoir Geology and Exploitation (Southwest Petroleum University), Chengdu, 610500, Sichuan, China; ^2^School of Geoscience and Technology, Southwest Petroleum University, Chengdu, 610500, Sichuan, China; ^3^Technology Center, Sichuan Coal Industry Group Limited Liability Company, Chengdu, 610091, Sichuan, China; ^4^School of Civil Engineering and Architecture, Southwest Petroleum University, Chengdu, 610500, Sichuan, China

##### Correspondence: Jianjun Liu - liujj0906@sina.com

*BMC Ecology* 2016,** 16(Suppl 2)**:02

In order to reveal the formation and release mechanisms of hydrogen sulfide(H_2_S) in Longtan coal mine, and provide theoretical guidance for safety production, based on the geochemistry theory and series experiments of H_2_S release, some important mechanism and laws were investigated. H_2_S in coal seam of Longtan mine was formed by the dissimilatory reduction of sulfate reducting bacteria to sulfate. The proportion of abiogenic gas is very small in coal mass, but during the coal mining process, H_2_S will be released because of the coal mass being broken and turned over; most H_2_S exists in the formation water. When coal mining causes mine water pouring out, the water flow disturbance will induce H_2_S dissolved in water releasing. The experiment results showed that with the water flow disturbance severity H_2_S release rate increases. The release rate in water flow upstream is bigger than that of downstream. When the water flows to a certain distance, no H_2_S releases from the water. Water pH value and temperature have some influence on the H_2_S release, the lower the pH value, the higher the temperature, the more hydrogen sulfide is easy to escape from the water.

## 03 Coral bleaching in the North Reef of China’s Xisha Islands in 2014

### Yuanchao Li^1^, Xinqing Zheng^2^, Shiquan Chen^1^, Haiqun Xie^1^

#### ^1^Hainan Academy of Ocean and Fisheries Sciences, Haihou 570100, China; ^2^Third Institute of Oceanography, State Oceanic Administration, Xiamen 361005, China

##### Correspondence: Yuanchao Li - lycouc@foxmail.com

*BMC Ecology* 2016,** 16(Suppl 2)**:03

The North Reef (NR) is located in China’s Xisha Islands in the Western Pacific, which contained successful coral reefs with a mean coverage that reached above 70 % before 2013 because there was little anthropogenic disturbance. However, an investigation in October 2014 showed that large-scale coral bleaching had occurred on the flat and that the bleaching rate exceeded 90 %. The bleached corals were primarily *Acropora formosa*. Some of the bleached branches had been overgrown with microalgae and became yellow, indicating that the corals had bleached and died several months prior, and cannot recover, even if the sea surface temperature (SST) declines below 30 °C. However, coral bleaching was not observed in the slope of the NR, found in deeper waters. We speculate that the large-scale coral bleaching in the NR flat was predominantly triggered by elevations in SST. According to data from the Space Science and Engineering Center, the SST from June to September 2014 was above 30 °C, which could easily cause coral to bleach, and hermatypic corals, especially *Acropora*, to do so when SSTs exceed this threshold. The NOAA also found that the degree-heating weeks (DHW) reached 4 °C-weeks in June 2014 and continued to rise, remaining above 8 °C-weeks from July to September 2014. It peaked in September 2014 at 12 °C-weeks. If the NR corals continue to be affected by high temperatures in 2015, as predicted by NOAA, the reef ecosystem will be further stressed, and large-scale coral mortality will be inevitable in the NR.

**Acknowledgements:** This work was supported by Xiamen Southern Oceanographic Center, Contract No: 14CZY037HJ11.

## 04 Soil chemical characteristics in a *Cunninghamia lanceolata* stand suffering from ice-snow damage

### Jie Li, Zhuomin Wang, Li Xue

#### College of Forestry and Landscape Architecture, South China Agricultural University, Guangzhou 510642, China

##### Correspondence: Li Xue - forxue@scau.edu.cn

*BMC Ecology* 2016,** 16(Suppl 2)**:04

Studies on soil chemical characteristics were conducted in a *Cunninghamia lanceolata* stand suffering from ice-snow damage in the north of Guangdong province from 2008 to 2011. The results showed that the pH of the upper soil layer (0–20 cm) and lower soil layer (20–40 cm) decreased with increasing time. The organic matter content of the upper soil layer increased significantly and then decreased very significantly, whereas that of lower layer decreased significantly. The change tendency of total N content was similar to the organic matter. Total P content of the two soil layers increased significantly, and then decreased very significantly. Total K of the two soil layers decreased with increasing time. Contents of Alkahi-hydrolyzable N, available P, available K of the upper soil layer were greater than those of the lower layer in each year. Urease activity of the upper soil layer decreased, whereas that of lower layer decreased, and then increased. Activities of phosphatase and catalase decreased and then increased.

## 05 Diversity of understory vegetation was under a *Cunninghamia lanceolata* stand suffering from ice-snow damage

### Zhiyang Lie, Weilong Huang, Tongtong Zhou, Li Xue

#### College of Forestry and Landscape Architecture, South China Agricultural University, Guangzhou 510642, China

##### Correspondence: Li Xue - forxue@scau.edu.cn

*BMC Ecology* 2016,** 16(Suppl 2)**:05

Diversity of understory vegetation was studied in a *Cunninghamia lanceolata* stand suffering from ice-snow damage in the north of Guangdong province from 2008 to 2011. The species in shrub layer increased, and most plants were shade-tolerance plants in the herbaceous plant layer from 2009. *Rubus rosaefolius* became the absolute dominant species in the shrub layer in 2010, and some new species, such as Aster ageratoides and *Dryopteris podophylla* appeared in herbaceous plant layer. The herbaceous plants occupied dominion position when the ice-snow damage occurred in 2008, and then many kinds of shade-tolerance shrubs invaded one after another. Richness index of the shrub layer under the *C. lanceolata* stand in 2009 was the lowest during 3-year study course. Simpson index in 2009 was larger than that in 2010, whereas Shannon-Wiener index was opposite. Richness index of the herbaceous plant layer under the stand in 2008 was compared to those in 2009 and 2010. The order of Simpson and Shannon-Wiener index indices of the 3 years were 2009, 2010, 2008. Therefore, the species diversity in 2009 was the highest and the stablest with well-distributed species.

## 06 The effect of water, nitrogen and harvesting time on yield and biomass allocation of *Helianthus tuberosus* L.

### Kai Gao, Tiexia Zhu

#### Inner Mongolia University for Nationalities, Tongliao, 028043, Inner Mongolia, China

##### Correspondence: Kai Gao - gaokai555@126.com

*BMC Ecology* 2016,** 16(Suppl 2)**:06

With the study on the effect of nitrogen fertilization (five fertilization levels), irrigation (including irrigation and without irrigation) and harvesting time (including September 25 and October 10) on height, biomass and the role of mater allocation of *Helianthus tuberosus* L. in north-eastern Inner Mongolia, China, the results showed that the height and yield of *H. tuberosus* L. were significantly improved by irrigation and nitrogen fertilization + irrigation (p < 0.05). Nitrogen fertilizer significantly influence height and yield during irrigation and achieved higher yield during addition of nitrogen (25–50 kg ha^−1^) (p < 0.01). On the contrary, it was not significantly influenced without irrigation. *Helianthus tuberosus* L. was early harvested when it was used for forage, and it was lately harvested when it was used for bio-ethanol and inulin; The root/shoot ratio and tuber mass ratio were significantly improved under fertilizer, water and nitrogen fertilizer + water condition (p < 0.05); The leaf mass ratio was remarkably improved under nitrogen fertilizer, water and nitrogen fertilizer + water condition during nutritional growth, and it did not indicate significant difference after nutritional growth; The growth, the yield, the water use efficiency and the yield of tuber were enhanced by the application of nitrogen fertilizer and water, and the using value for energy was improved by nitrogen fertilization and irrigation.

**Acknowledgements:** The National Natural Science Foundation project, Project Number 31560672.

## 07 The vertical variation of microbial communities in the sediment of sea cucumber pond

### Weiteng Mao^1^, Pei Yu^1^, Xinfang Lv^1^, Yunchun Li^1,2^

#### ^1^Marine College, Shandong University, Weihai 264209, China; ^2^Institute of Ecology and Biodiversity, Shandong University, Jinan 250100, China

##### Correspondence: Xinfang Lv - lvxinfang@hotmail.com

*BMC Ecology* 2016,** 16(Suppl 2)**:07

The sediment of aquaculture pond has important influence on the water quality and the health of sea cucumber (*Apostichopus japonicas* Selenka). We collected the sediment in a sea cucumber pond which is located in Rongcheng and divided it into 9 samples named from L1 (0–3 cm) to L9 (24–27 cm). The environmental factors of all samples have been measured, with the 16SrDNA sequencing by the illumina miseq to detect the microbial communities in the samples L1, L2, L6 and L9. The sediment has the characteristics of high-organic-matter, high-nitrogen, high-sulfur and low-phosphate. The dominant bacteria are the phylum proteobacteria, acidobacteria, bacteroidetes, chloroflexi, planctomycetes, gemmatimonadetes and nitrospirae. The content of bacteroidetes tends to decrease with the increase of sediment depth, while the contents of chloroflexi, gemmatimonadetes and nitrospirae show the opposite tendency. PCA analysis and SPSS correlation analysis show that the microbial communities are mainly affected by phosphorus and salinity. The abundant sequences of probiotics were detected in our study such as photosynthetic bacteria (PSB), nitrifying bacteria, *bacillus* and *bdellovibrio*. Our study takes a new look at the sterilization in the aquaculture of sea cucumber and provides theoretical guidance for the exploitation and application of probiotics.

## 08 Indicating significances of alpine plant functional groups to environmental change

### Peixi Su, Rui Shi

#### Cold and Arid Regions Environmental and Engineering Research Institute, Key Laboratory of Land Surface Process and Climate Change in Cold and Arid Regions, Chinese Academy of Sciences, Lanzhou 730000, China

##### Correspondence: Peixi Su - supx@lzb.ac.cn

*BMC Ecology* 2016,** 16(Suppl 2)**:08

Alpine plants on the Zoige Plateau in the Eastern Qinghai-Tibetan Plateau of China were divided into five functional groups based on hydro-ecological characteristics and δ^13^C value using an ecological investigation employing horizontal and vertical belt transects (listed here from dry to wet habitats): xeromesophytes, mesophytes, hygromesophytes, hygrophytes and hydrophytes. Plants were also divided by physiognomic life form and classification features into six groups: tree, shrub, grass, sedge, weed and aquatic species. Based on Raunkiaer’s classification system, which classifies plant adaptation into harsh and cold environments, the alpine plants were divided into five groups: phanerophytes, chamaephytes, hemicryptophytes, geophytes, and therophytes. Hemicryptophytes and geophytes accounted for 66 % of the total alpine plant species on the Zoige Plateau, of which perennial herbaceous plant species take 89 % of the total number of herbaceous plant species. In alpine plant community regressive succession with water environment deteriorating, from wetland, swampy meadow, wet meadow, dry meadow, to degraded meadow phase, hygrophytes were replaced by mesophytes and xeromesophytes. Over time increasingly abundant dominant grass species displaced dominant sedge species with less abundance. Dicotyledonous weed plants were dominant in highly degraded meadow, and increased in abundance during succession. Succession tended to move from alpine meadow to alpine shrubby meadow.

## 09 Research on the daily CH_4_ fluxes of soil in summer mangrove community at Yingluogang of Guangxi, China

### Yong Jiang^1,2^, Xingbin Chen^1,2^, Shichu liang^1,2^

#### ^1^College of Life Science, Guangxi Normal University, Guilin, 541004, Guangxi China; ^2^Key Laboratory of Ecology of Rare and Endangered Species and Environmental Protection (Guangxi Normal University), Ministry of Education, Guilin 541004, China

##### Correspondence: Shichu liang - gxlsc@sina.com

*BMC Ecology* 2016,** 16(Suppl 2)**:09

Mangrove ecosystems are known sources for methane (CH_4_) having very high global warming potential on global change. In order to better understand CH_4_, dynamics will affect global change in wetland system. We have quantified the daily CH_4_ fluxes for various species within mangrove wetland communities (*Kandelia candel*, *Bruguiera gymnorhiza*, *Rhizophora stylosa* and *Aegiceras corniculata* communities) in southwest China. Additionally, we evaluated the influence of different environmental factors (air temperature, soil temperature, soil pH and salinity) on daily CH_4_ fluxes. According to the measurements from a portable automated flux system, the mean fluxes of the daily CH_4_ were the lowest in *B. gymnorhiza* community, the higher in *Aegiceras corniculatum* and *A. corniculata* community, and the highest in the *K. candel* community. Variation in fluxes of the daily CH_4_ was mainly determined by soil pH among the four communities in the summer. Daily CH_4_ fluxes were positively related to soil pH in *R. stylosa* community and negatively related to soil pH in *A. corniculata* community; however, pH was uncorrelated with CH_4_ fluxes in either *K. candel* or *B. gymnorhiza* communities. We draw conclusion that significant differences in daily CH_4_ fluxes are linked to soil pH in several mangrove wetland communities.

**Acknowledgements:** This work was supported by fund for key research project of natural science foundation of Guangxi (2012 GXNSFEA 053001).

## 10 Beach protection structures in middle and lower reaches of Yangtze River

### Junfeng Xu

#### Tianjin Research Institute for Water Transport Engineering, Key Laboratory of Engineering Sediment, Ministry of Transport, Tanggu, Tianjin 300456, China

##### Correspondence: Junfeng Xu - 13920354156@139.com

*BMC Ecology* 2016,** 16(Suppl 2)**:10

Lots of beach protection measures have been taken in the middle and lower Yangtze River, such as fish-born dikes, soft mattresses, mesh pads and tetrahedron-like penetrating frames. These structures of beach protection can realize basic functions. But for the ecological protection of the river, there is a certain distance. The existing river ecological engineering researches mainly include the ecological environmental protection building materials and vegetation beach protection technology. Based on the practical effect, the commonly used traditional building material has a higher maintenance cost, which does not have the ecological function and poor landscape effect. Through the model test, the most easily damaged parts and the main failure reasons of these structures and measures to prevent damage and relevant restoration measures are also analyzed in the paper. The effect of a new kind of beach protection structure applied in Middle and Lower Reaches of Yangtze River is analyzed and the adaptability of this new structure is concluded. It serves as reference for similar projects.

**Acknowledgements:** This research was supported by funding from The central level, scientific research institutes for basic R & D special fund business (TKS150102).

## 11 Social responsibility management of large dam project with owner as core: an empirical case in China

### Jiwei Zhu^1^, Zhao Zhai^2^, Linan Zhou^1^, Yun Le^2^, Li Cao^3^

#### ^1^State Key Laboratory of Ecological Water Conservancy in the Northwest Arid Area, Xi’an University of Technology, Xi’an, 710048, China; ^2^Research Institute of Complex Engineering and Management, Tongji university, Shanghai 200092, China; ^3^China Three Gorges Corporation, Beijing 100038, China

##### Correspondence: Jiwei Zhu - zhujiwei@xaut.edu.cn

*BMC Ecology* 2016,** 16(Suppl 2)**:11

As major infrastructure project, the large dam project (LDP) has functions such as flood control, power generation, irrigation, water supply and so on. LDP could lead to a series of ecological and social effects due to the potential inundation, resettlement, and change of hydrological regime. How to reduce its negative effects and ensure public interests is the key issue to promote LDP management and sustainable development. The owner as an investor is the core role to undertake the society responsibility of LDP. Based on the analysis of relationship among various stakeholders, we propose a management framework of social responsibility of large dam project (LDP-SR), which includes three dimensions: responsibility, organization and project life-cycle. We also establish the evaluation index system of LDP-SR for the owner by adopting corporate social responsibility (CSR). Taking the Three Gorges Project in China as an example, the social responsibility performance is evaluated by using the AHP-MF model. The research findings contribute to knowledge of stakeholder management of LDP-SR and provide a solution for the owner of LDP to fulfill social responsibility and promote sustainable development.

## 12 The ecological function value assessment analysis of urban waterfront

### Jiwei Zhu^1^, Ping Lu^1^, Zhiqian Huang^2^, Bei Zhou^1^

#### ^1^State Key Laboratory of Ecological Water Conservancy in the Northwest Arid Area, Xi’an University of Technology, Xi’an 710048, China; ^2^Ji’nan City Construction Investment Group Corporation Limited, Ji’nan 250101, China

##### Correspondence: Jiwei Zhu - zhujiwei@xaut.edu.cn

*BMC Ecology* 2016,** 16(Suppl 2)**:12

Taking unbar waterfront in Xi’an—Chan Ba wetland as an example, this paper establishes the ecological function value evaluation index system to study on function value of urban waterfront ecosystem. This paper calculates and researches ecological function value by using some mathematic and economic method, such as the market value method, carbon tax method,the afforestation cost method, the alternative cost method, the shadow engineering method, ecological value method, and traveling cost method. Through calculation, the ecological function value of Chan-Ba wetland is ¥3.87 × 10^8^, in which the ratio of material production function, ecological regulation function and social service function value was 3.65, 38.59, 57.76 %, respectively. The value of Chan-Ba wetland is a rational combination between environmental protection and function of science, education and entertainment. Urban waterfront ecological function value has an important position in the urban economic and social development and ecological construction. Only when reasonable development and protection of the urban waterfront ecological environment can the effective utilization of resources be achieved and sustainable development of urban economy be ensured.

## 13 Mechanism of diurnal osmotic potential changes and adjustment of three *Caragana* species in different habitats of the Inner Mongolia Plateau

### Zhirong Zheng^1, 2^, Shihai Lv^2^, Chaoyang Feng^2^

#### ^1^College of Life Science, Nankai University, Tianjin 300071, People’s Republic of China; ^2^State Environmental Protection Key Laboratory of Regional Eco-pro**c**ess and Function Assessment, Chinese Research Academy of Environmental Sciences, Beijing 100012, People’s Republic of China

##### Correspondence: Chaoyang Feng - fengchy@craes.org.cn

*BMC Ecology* 2016,** 16(Suppl 2)**:13

Plants belonging to the genus *Caragana* are selected as preferred legume shrubs for water conservation and sand fixation in the arid and semi-arid areas of the Mongolian Plateau. The distributions of three *Caragana* species (*Caragana microphylla*, *Caragana davazamcii*, and *Caragana korshinskii*) exhibit remarkable regional-level distribution characteristics, whereby they replace each other along a longitudinal water gradient from east (semi-arid habitat) to west (arid habitat). We investigated the cell water relations for this substituted distribution by: (1) comparing diurnal variation in osmotic potential, and (2) analyzing the relative contributions of three regulatory mechanisms to diurnal variation in osmotic potential. We found that dehydration and intracellular net solute accumulation were the main regulatory mechanisms used by all three *Caragana* species to adjust diurnal variation in osmotic potential. The relative contribution of dehydration to osmotic potential changes of *C. microphylla*, *C. davazamcii* and *C. korshinskii* was 76, 56, and 52 %, respectively, while that of net solute accumulation was 38, 39, and 34 %, respectively. As drought conditions increased, the regulating capacity of cell water gradually increased from *C. microphylla* to *C. davazamcii* and *C. korshinskii*, forming the stable physiological basis for their substituted distribution on the Mongolian Plateau.

## 14 Biodiversity of phytoplankton and environmental influences analysis of Longfeng Wetland, China

### Nan Song, Yan Liu, Xinxin Lu, Yawen Fan

#### College of Life Science and Technology, Harbin Normal University, Harbin, Heilongjiang, People’s Republic of China

##### Correspondence: Yawen Fan - fanyaw@163.com

*BMC Ecology* 2016,** 16(Suppl 2)**:14

The water quality of Longfeng Wetland was surveyed in 2011 to investigate the relationship between phytoplankton and environmental variables in the present study. Data of environmental conditions of five sites were different, the highest quality of the water was observed at site 2. A total of 99 genera of phytoplankton were identified, which belonged to 8 phyla and 47 families. Cyanophyta was absolutely dominant (approximately 47.56 % of total), and Bacillariophyta represented 18.05 % of total, showing that Longfeng Wetland was a Cyanophyta–Bacillariophyta predominant wetland. The highest number of genera of phytoplankton community was present in places with turbulent water (site 4) and scenic spot (site 2). The multimetric indexes indicated that this place was less influenced from August to September. The most dominant species were *Nitzschia palea* and Cyclotella *meneghiniana* in Longfeng Wetland, which belonged to Bacillariophyta. The canonical correspondence analysis (CCA) showed that the SpCond, TN, BOD5, and CODcr were the most effective environmental factors and having the greatest influence on the dominant species. In addition, pH and DO had considerable influence on *Scenedesmus dimorphus*. Our findings may imply that the biomass of *N. palea* and *S. dimorphus* can be used as biological quality indicators in wetland.

**Acknowledgements:** This work were supported by NSFC(31270250, 31470308) and the Graduate Innovative Research Projects of Heilongjiang Province (No. YJSCX2011-412HLJ).

## 15 The effect of human activities on culturable soil microbes from Yaoquan Mountain in Wudalianchi, China

### Jianfei Guan^1^, Jihua Wang^2^, Xueping Zhang^1^

#### ^1^College of Geographical Science, Harbin Normal University, Harbin, Heilongjiang, 150025, China; ^2^College of Life Science and Technology, Harbin Normal University, Harbin, 150025, Heilongjiang, China

##### Correspondence: Jihua Wang - wangjihua333@hotmail.com

*BMC Ecology* 2016,** 16(Suppl 2)**:15

The effect of human activities on the soil microbial community structure and the relationships among microbial community members, soil chemical properties, and enzyme activity levels were assessed using soil samples from Yaoquan Mountain in Wudalianchi, China. Soil cultures revealed *Bacillus*, *Streptomyces*, *Aspergillus*, and *Penicillium* which were common microbial species in Yaoquan Mountain soil samples. The degree of human disturbance was negatively correlated with disturbance in the microbial community composition, so that quantitative changes in the representation of actinomycetes and fungi were more sensitive to human activities than that of bacteria. The survey also revealed spatial heterogeneity in soil microorganisms. Within a given sampling area of the mountain, the separation frequency of some strains changed clearly with the increases in disturbance. Accordingly, *Enterobacter* and *Penicillium* may be used as indicative bacteria for evaluating human disturbance on mountaintops, while *Aspergillus*, *Penicillium*, *Lactobacillus*, and *Streptomyces* are better suited for evaluating human disturbance on mountainsides. Soil organic matter, total nitrogen, catalase, and cellulase levels each exhibited extremely significant correlations with the level of human disturbance. Organic matter, catalase activity, cellulase activity, and pH greatly influenced the microbial community structure. By revealing the quantity and structure of microorganisms and their relationship with environmental factors, this research has characterized the effect of human disturbance on Yaoquan Mountain.

